# Stressful environments can indirectly select for increased longevity

**DOI:** 10.1002/ece3.1013

**Published:** 2014-03-10

**Authors:** Fiona R Savory, Timothy G Benton, Varun Varma, Ian A Hope, Steven M Sait

**Affiliations:** 1 Faculty of Biological Sciences, School of Biology, University of LeedsLeeds, LS2 9JT, U.K; 2 National Centre for Biological Sciences, TATA Institute of Fundamental ResearchBangalore, 560 065, India

**Keywords:** *Caenorhabditis elegans*, fitness, longevity, stress resistance, trade-offs

## Abstract

Longevity is modulated by a range of conserved genes in eukaryotes, but it is unclear how variation in these genes contributes to the evolution of longevity in nature. Mutations that increase life span in model organisms typically induce trade-offs which lead to a net reduction in fitness, suggesting that such mutations are unlikely to become established in natural populations. However, the fitness consequences of manipulating longevity have rarely been assessed in heterogeneous environments, in which stressful conditions are encountered. Using laboratory selection experiments, we demonstrate that long-lived, stress-resistant *Caenorhabditis elegans age-1(hx546)* mutants have higher fitness than the wild-type genotype if mixed genotype populations are periodically exposed to high temperatures when food is not limited. We further establish, using stochastic population projection models, that the *age-1(hx546)* mutant allele can confer a selective advantage if temperature stress is encountered when food availability also varies over time. Our results indicate that heterogeneity in environmental stress may lead to altered allele frequencies over ecological timescales and indirectly drive the evolution of longevity. This has important implications for understanding the evolution of life-history strategies.

## Introduction

Mutations that increase life span in model organisms typically incur trade-offs, such as delayed maturity or low fecundity, which reduce fitness relative to wild-type alleles (Jenkins et al. 2004; Van Voorhies et al. 2006). This is consistent with evolutionary theories of ageing (Williams 1957; Kirkwood 1977) and suggests that mutations that promote longevity are unlikely to become established in wild populations. However, the nature and magnitude of trade-offs among life-history traits can differ depending upon the environmental context in which they are observed (Sgrò and Hoffmann 2004). For example, long-lived mutants have been identified which have equal fitness to wild-type genotypes in favorable environments, but the same mutants display trade-offs under nutrient-limited conditions (Walker et al. 2000; Marden et al. 2003; Delaney et al. 2011). Such context-dependent trade-offs provide important examples of how mutations that increase life span can incur fitness costs in ecologically relevant conditions. Nevertheless, given the extensive variation in life span that is observed within and between species in nature, there must be situations in which mutations that promote longevity can be beneficial.

With the exception of species that exhibit grand maternal care, selection does not act upon postreproductive life span (Williams 1957; Hamilton 1966; Lahdenperä et al. 2004; Foster et al. 2012), and longevity is expected to evolve in response to selection on associated traits. Environmental stress is predicted to be an important driver of the evolution of longer life spans in nature due to overlapping requirements for protection and repair of somatic molecules and cells (Parsons 1995; Kenyon 2005). Selection acting upon genetic variation for stress resistance could thus have important consequences for the evolution of longevity in natural populations, and this is especially relevant given expected changes in global climate, more extreme environmental events, and additional anthropogenic pressures. The role of stress in the evolution of increased longevity has been tested with artificial selection experiments, but support for the prediction has been inconsistent (White et al. 1970; Rose et al. 1992; Hoffmann and Parsons 1993; Harshman et al. 1999; Pijpe et al. 2008). Surprisingly, although many mutations that extend life span disrupt conserved signal transduction pathways which regulate responses to environmental stress (Kenyon 2005), the evolutionary fate of such mutations has not been assessed in adverse abiotic conditions.

In *Caenorhabditis elegans*, the conserved insulin/IGF-1 signaling (IIS) pathway modulates development, metabolism, stress resistance, and longevity, in response to environmental change, by regulating the activity of the FOXO transcription factor DAF-16 (Ogg et al. 1997). Mutants which are defective for components of the IIS pathway, such as DAF-2, the insulin/IGF-1 receptor homologue, or AGE-1, the phosphatidylinositol 3-kinase (PI3K) catalytic subunit homologue, are long-lived (Friedman and Johnson 1988; Kenyon et al. 1993) and display increased resistance to various forms of stress which may be encountered in nature, such as temperature extremes (Lithgow et al. 1994, 1995; Savory et al. 2011) and pathogen infections (Garsin et al. 2003).

Loss-of-function mutations in the IIS pathway can lead to life-history trade-offs by inducing developmental arrest in the stress-resistant dauer larval stage in conditions which are permissive for wild-type growth and reproduction (Malone et al. 1996; Morris et al. 1996; Gems et al. 1998). Even under conditions which do not induce dauer arrest in IIS mutants, longevity is typically traded off against other life-history traits such as fecundity, leading to a net reduction in fitness (Gems et al. 1998; Tissenbaum and Ruvkun 1998; Jenkins et al. 2004; Van Voorhies et al. 2006). However, long-lived, stress-resistant mutants bearing the partial loss-of-function *age-1(hx546)* allele have equal fitness to the wild-type genotype at favorable growth temperatures when excess food is available (Walker et al. 2000). These mutants arrest in the dauer stage at moderately high temperatures (27°C) which do not induce dauer formation in wild-type individuals (Malone et al. 1996; although see Ailion and Thomas 2000) and display fitness costs when mixed genotype populations are exposed to cycles of starvation (Walker et al. 2000). Although this suggests that the *age-1*(*hx546*) mutant allele could be selected against in heterogeneous environments that include fluctuations in food availability and temperature, the wild-type *age-1(+)* allele may not always confer a selective advantage because *age-1*(*hx546*) mutants are more likely to survive in certain stressful environments (Kenyon 2005).

To test the role of environmental stress in the evolution of longevity, we used competition assays to compare how selection acts upon the *C. elegans age-1(hx546)* mutant and wild-type *age-1(+)* alleles under a variety of environmental conditions. These included different nutritional environments and periodic exposure to intermediate (27°C) or high (30°C) temperature stress. We also examined gene-by-environment interactions to determine which life-history traits contribute to observed differences in fitness between the two genotypes, and used population projection models to compare fitness in more stochastic environments.

## Materials and Methods

### Strains and culture conditions

The wild-type (N2 Bristol) and homozygous *age-1(hx546)* (TJ1052) genotypes were obtained from the Caenorhabditis Genetics Centre, University of Minnesota. Populations were cultured in Petri dishes containing nematode growth media (NGM) with 10 *μ*g/mL nystatin and 50 *μ*g/mL streptomycin. *Escherichia coli* (HB101) was provided as a food source. Populations were maintained at 20°C except when stated otherwise.

### Laboratory selection experiments

To obtain age-synchronized wild-type and *age-1(hx546)* mutant larvae, eggs from young adult hermaphrodites maintained with ad lib food were transferred to fresh plates, and newly hatched larvae were collected within a 1–2-h period. Mixed genotype populations were then initiated with two wild-type and two *age-1(hx546)* mutant hermaphrodites when the age-synchronized larvae had reached the fourth larval stage (day 0). Populations were allocated to unlimited food or limited food treatments, which were achieved by maintaining populations at low densities or high densities, respectively. In *C. elegans*, population density is perceived via concentrations of a dauer pheromone which is constitutively produced by all worms (Golden and Riddle 1982). We are not aware of any empirical data suggesting that wild-type worms and *age-1(hx546)* mutants respond differently to this pheromone. Therefore, we considered that differences that arose between the genotypes under these treatments were more likely to reflect different physiological responses to food availability rather than to population density per se. To maintain the required differences in food availability, populations were transferred to a new food source every second day in M9 buffer solution under sterile conditions. For populations with unlimited food, this began on day 3, when a prominent bacterial lawn was visible, and approximately 20% of each original population (containing mixed stage individuals) was transferred. For populations with limited food, this began on day 5, when food had become scarce or had been depleted, and approximately 90% of each original population (containing mixed stage individuals) was transferred. For each food treatment, four replicate populations containing mixed stage individuals were constantly maintained at 20°C (a favorable growth temperature), four were periodically exposed to 27°C (moderate thermal stress), and four were periodically exposed to 30°C (intense thermal stress). Thermal stress treatments were imposed for 24 h on days 6, 12, and 18 (Fig. S1).

To monitor allele frequencies, approximately 50 eggs from each population were transferred to 27°C on days 6, 12, and 18 before temperature treatments commenced, and on day 24 (Fig. S1). As *age-1(hx546)* mutants arrest in the dauer larval stage at 27°C whilst wild-type worms develop into adults (Malone et al. 1996)*,* genotype frequencies were determined by counting the dauers and adults derived from each population (Walker et al. 2000). Wild-type larvae occasionally arrest as transient dauers at 27°C, but rapidly resume development at this temperature (Ailion and Thomas 2000). To account for this, we allowed 3 days for development before counting the dauers and adults derived from each population, and eggs of known genotype were always tested in parallel to confirm the reliability of the assay. To ensure that sufficient eggs were obtained from populations with limited food availability, eggs were collected less than 24 h after food had been provided. The experiment was repeated in two separate temporal blocks. The number of generations which elapsed during the experimental period was not explicitly quantified. However, this would have varied among treatment groups, with a maximum of nine generations for populations maintained with unlimited food at 20°C. Populations were regularly examined for the presence of males, but these were rarely observed and mating was not considered to influence the results.

A generalized linear mixed effects model with a binomial error distribution and a logit link function was fitted to the data using the penalized quasi-likelihood (PQL) method in R version 2.14.1 (R Core Team 2013). An auto-correlation function was included to account for repeated measures on the same populations over time. The model contained all explanatory variables (day, temperature, food availability) and their interactions, and treatments were nested within a random effects term to account for variation between temporally replicated blocks.

### Gene-by-environment interactions

Survival, times to maturity, and fecundity data were collected to examine gene-by-environment interactions in *age-1(hx546)* mutants, and the wild-type genotype after fed and starved larvae at different stages of development were exposed to different temperature treatments. Larvae maintained with ad lib food were age-synchronized, as described above, immediately before thermal stress treatments commenced (L1s) or 24 h in advance (L3s). Larvae which had arrested development in the L1 diapause stage (this stage is formed immediately after eggs hatch in the absence of food) or the dauer stage were obtained from populations which had been starved for approximately 24 h before the thermal stress treatments were implemented. These remained starved during the temperature treatments, but were provided with food immediately after the thermal stress period, so that development could resume. Fed and starved larvae were maintained at 20°C, or were transferred to 27°C or 30°C for 24 h before being shifted back to 20°C, then were observed until death or until reproduction ceased. Whilst poststress survival and poststress times to maturity were assessed in five temporally replicated blocks, poststress fecundity data were collected over three temporally replicated blocks (sample sizes are presented in Table S1).

Survival status was visually assessed after the thermal stress period then intermittently until maturity. If survival status was unclear, worms were gently touched with a platinum wire to stimulate a response. Differences in survival at 30°C were compared between genotypes and between fed L1s and L3s using a generalized linear model with a quasi-binomial error distribution and a logit link function. The minimum adequate model contained only genotype as an explanatory variable.

To monitor poststress times to maturity, individuals who had reached the final larval stage (L4) were examined at 1–2-h intervals until egg-laying commenced. As times to maturity were monitored only after the stress treatments were terminated, and were dependent upon the stage which had been attained before stress treatments were implemented, each stage was analyzed separately. Poststress times to maturity were compared between genotypes and among temperature treatments with generalized linear mixed effects models. The models, which were fitted using the PQL method, had gamma error distributions and random effects terms to account for variation among temporally replicated blocks.

To monitor fecundity, adults were regularly transferred to new plates until reproduction had ceased and then the offspring on each plate were counted. When the number of offspring on a plate was relatively low, counting was performed simply by eye. When many offspring were present on a plate, worms were removed individually using a platinum pick. Fecundity was compared between genotypes and among stages and treatments using a linear mixed effects model. The model contained all explanatory variables (genotype, stage, and temperature) and their interactions and a random effects term to account for temporal blocks.

### Simulating population growth in stochastic environments

Population projection models were used to simulate population growth for homozygous *age-1(hx546)* mutants and the wild-type genotype under a variety of stochastic environmental conditions. These reflected environments with (1) fluctuating food availability, (2) fluctuating food availability and low frequencies of thermal stress, (3) fluctuating food availability and intermediate frequencies of thermal stress, or (4) fluctuating food availability and high frequencies of thermal stress. For simplicity, it was assumed that population dynamics were not subjected to density-dependent regulation and that no effects of the maternal environment arose in subsequent generations. The population projection models were not spatially explicit. However, population vectors were reduced to contain only dauer larvae, the principal *C. elegans* dispersal stage (Cassada and Russell 1975), after each period of starvation to reflect movement away from unfavorable environments.

#### Construction of population projection matrices

Fourteen population projection matrices were created for each genotype in R version 2.14.1 to simulate population growth under different environmental conditions. The conditions included favorable environments (1 matrix), starvation (2 matrices), recovery from starvation (2 matrices), unlimited food and 27°C (1 matrix), recovery from unlimited food and 27°C (2 matrices), unlimited food and 30°C (1 matrix), recovery from unlimited food and 30°C (3 matrices), starvation and 27°C (1 matrix), and starvation and 30°C (1 matrix). Each matrix contained 22 stage/age classes which were simplified to reflect the possible life-history transitions and differences in fecundity which could arise when populations were projected in 1-day increments under different environmental conditions (Fig. S2; Table S2). It was necessary to include all of these stage/age classes to account for alternative routes of development which are selected and differences in poststress fecundity which arise depending on the environmental conditions which are experienced at different times in life. A description of the major events for each projection matrix is provided in Table S2. Matrix parameters (i.e., probabilities of survival from one age/stage class to the next and the expected number of offspring produced at each reproductive age) were obtained for each genotype and environmental condition using mean survival, time to maturity and fecundity values (Table S1). Age-specific fecundity parameters were extrapolated from the lifetime fecundity values using reproductive schedules from a pilot study.

#### Randomization of environmental states

Four sets of simulations were implemented using R version 2.14.1: resource fluctuation with no thermal stress, resource fluctuation with low frequencies of thermal stress, resource fluctuation with intermediate frequencies of thermal stress, and resource fluctuation with high frequencies of thermal stress. Vectors were created containing environmental states which could be encountered when populations had previously been projected in favorable conditions or starvation conditions, or when populations were recovering from starvation or thermal stress. Probability distributions were created for each vector to control the frequency at which different states could arise. After initial projections in favorable conditions (×10 time steps), environmental states were selected at random from the appropriate vector at a frequency determined by the relevant probability distribution. To account for life-history transitions which arise during starvation or recovery from starvation or thermal stress, matrices were placed in a specific order when necessary (Fig. S3; Table S2). Randomized matrix sequences were identical for wild-type and *age-1(hx546)* mutant populations except during recovery from exposure to 30°C with unlimited food (Fig. S3; Table S2).

#### Projecting populations in stochastic conditions

Starting population vectors for each genotype contained 999 individuals at a stable age distribution (determined from the right eigenvectors of the matrices for favorable conditions). New population vectors were derived at each subsequent time step according to the equation *N*_*t* + 1_ = MN_*t*_, where M is the projection matrix at time *t* and *N*_t_ is the population vector at time *t* (Benton and Grant 1996). After each starvation period, the population vector for each genotype was altered to contain only a proportion of the dauer larvae which were present in the previous population vector. Proportions varied at random from 0.1% to 1%, but were always the same for both genotypes for a given starvation period. The range of proportions was based on the assumption that, in natural populations, only a small proportion of dauer larvae will successfully disperse to and exploit a new patch of food. About 1000 Monte Carlo simulations were implemented for each set of randomized matrix sequences. The stochastic population growth rate (*λ*_s_) was determined for each genotype using the equation log *λ*_s_ = 1/1000 log (*N*_1000_−*N*_0_), where *N*_1000_ is the mean population size after 1000 time steps and *N*_0_ is the initial population size (Benton and Grant 1996). *λ*_s_ values were then used to determine the relative fitness of *age-1(hx546)* mutants for each set of simulations.

## Results

### The *age-1(hx546)* mutant allele confers a selective advantage in stressful environments when food is available

**Figure 1:**
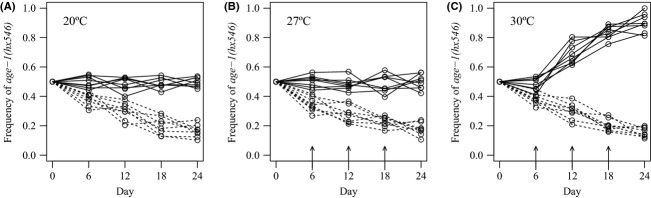
*age-1(hx546)* mutants have higher fitness than the wild-type genotype if periodically exposed to intense thermal stress when food is available. Temporal changes in frequencies of the *age-1(hx546)* allele, relative to the wild-type *age-1(+)* allele, in populations with unlimited (solid lines) and limited (broken lines) food which were (A) maintained at 20°C, (B) periodically exposed to 27°C, or (C) periodically exposed to 30°C. Arrows indicate days on which 24 h thermal stress treatments (27°C or 30°C) were implemented. Data are presented from two separate experimental blocks in which four replicate populations were exposed to each treatment.

To test the role of stress in the evolution of longevity, we established mixed genotype populations with equal proportions of wild-type and *age-1(hx546)* mutant individuals, and monitored temporal changes in allele frequencies when populations with unlimited or limited food availability were maintained at a favorable growth temperature (20°C) or were periodically exposed to moderate (27°C) or intense (30°C) thermal stress. When mixed genotype populations were maintained at 20°C with unlimited food, allele frequencies remained relatively constant throughout the experimental period (Fig. 1A). However, when populations with limited food were maintained at this temperature, frequencies of the *age-1(hx546)* allele declined over time (*t* = −17.25, *P *< 0.001; Fig. 1A). These results reflect previous findings (Walker et al. 2000) and suggest that the *age-1(hx546)* allele disrupts the optimal response to nutritional stress. Although the *age-1(hx546)* allele induces developmental arrest at 27°C (Malone et al. 1996), no significant differences were observed in populations with either unlimited or limited food that were periodically exposed to this temperature compared to the equivalent populations maintained at 20°C (Fig. 1B). Notably, the *age-1(hx546)* allele rapidly increased in frequency when populations with unlimited food were periodically exposed to 30°C (*t* = 12.69, *P *< 0.001; Fig. 1C), demonstrating that the *age-1(hx546)* mutant life-history strategy confers a selective advantage under these conditions. Nevertheless, when populations with limited food were periodically exposed to 30°C, temporal changes in *age-1(hx546)* allele frequencies were not significantly different from those observed in the equivalent populations maintained at 20°C (Fig. 1C).

### Gene-by-environment interactions underlie the outcome of selection

**Figure 2:**
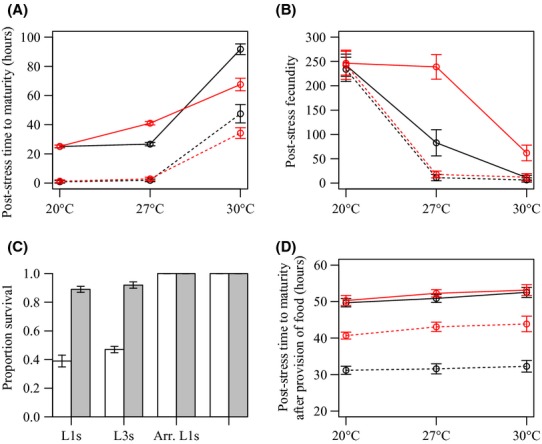
Gene-by-environment interactions. Life-history responses to thermal stress in wild-type individuals (black lines in A, B, and D; white bars in C) and *age-1 (hx456)* mutants (red lines in A, B, and D; gray bars in C). Solid lines represent L1s maintained with ad lib food (A and B) or arrested L1s (D), and broken lines represent L3s maintained with ad lib food (A and B) or dauers (D). Figures represent (A) mean times to maturity after the thermal stress period in individuals maintained with ad lib food availability during development, (B) mean fecundity (number of offspring per adult) after the thermal stress period in individuals maintained with ad lib food availability during development, (C) mean proportions of wild-type and *age-1(hx546)* mutant individuals which survived following exposure to 30°C during the L1, L3, arrested L1 (Arr.L1) and dauer larval stages, and (D) mean times to maturity after the thermal stress period and after food had been provided in individuals that had arrested development in the L1 diapause stage or the dauer stage. In all plots, error bars represent standard errors of the means. Time to maturity and survival data are presented for five temporally replicated blocks, and fecundity data are presented for three temporally replicated blocks.

Understanding how life-history responses to starvation and thermal stress vary between *age-1(hx546)* mutant and wild-type individuals could reveal why fitness differences arose between the two genotypes under specific environmental conditions. Therefore, we examined gene-by-environment interactions for several life-history traits after individuals of each genotype had been maintained at 20°C or exposed to moderate (27°C) or intense (30°C) thermal stress for 24 h during different stages of development. Consistent with the laboratory selection experiments, no significant differences were observed between the two genotypes in poststress survival, poststress times to maturity, or poststress fecundity when individuals were maintained at 20°C with ad lib food (Fig. 2A,B; Table S1). Similarly, no significant differences in these traits were observed between the genotypes when individuals maintained with ad lib food were exposed to 27°C during the L3 larval stage (Fig. 2A,B; Table S1). However, when individuals with ad lib food had been exposed to 27°C during the L1 stage, maturity was delayed in *age-1(hx546)* mutants relative to wild-type worms (*t* = −20.6, *P *< 0.001; Fig. 2A; Table S1), but fecundity was considerably higher (*t* = 17.07, *P *< 0.001; Fig. 2B; Table S1). These gene-by-environment interactions are likely to have arisen because *age-1(hx546)* larvae arrest development in the dauer stage at 27°C whilst wild-type worms do not (Malone et al. 1996). The effects of delayed maturity in *age-1(hx546)* mutants and reduced fecundity in the wild type may counteract one another, explaining why no difference in fitness was observed between the genotypes when mixed genotype populations were periodically exposed to 27°C (Fig. 1B).

Survival was considerably higher in *age-1(hx546)* mutants than the wild-type genotype when individuals maintained with ad lib food had been exposed to 30°C during the L1 and L3 larval stages (*F* = 197.68, *P *< 0.001; Fig. 2C; Table S1). Although times to maturity were highly variable in both genotypes, *age-1(hx546)* mutants also attained maturity more rapidly than the wild type under these conditions (L1s: *t* = 8.04, *P *< 0.001; L3s: *t* = 8.88, *P *< 0.001; Fig. 2A; Table S1), and exhibited higher fecundity when exposed to 30°C during the L1 stage (*t* = 4.80, *P *< 0.001; Fig. 2B; Table S1). These differences in plastic responses to thermal stress are likely to account for the variation in fitness observed between the genotypes when mixed genotype populations maintained with unlimited food were periodically exposed to 30°C (Fig. 1C).

When larvae were starved prior to and during the thermal stress treatments, no mortality was observed (Fig. 2C; Table S1) and no significant differences in fecundity arose between *age-1(hx546)* mutants and the wild-type genotype (Table S1). Furthermore, when starved larvae were exposed to high temperatures, no significant differences in growth rates were observed between the genotypes after food was replenished relative to those observed when no thermal stress was imposed (Fig. 2D; Table S1). The most striking difference observed between the two genotypes in starvation conditions was the delay in maturity which occurred in *age-1(hx546)* mutants after larvae had arrested development in the dauer stage (*t* = −26.79, *P *< 0.001; Fig. 2D; Table S1). As the ability to rapidly resume development when conditions permit is likely to be under strong selection, these observations may explain why frequencies of the *age-1(hx546)* allele consistently declined in mixed genotype populations with limited food even when thermal stress was imposed (Fig. 1A–C).

### The *age-1(hx546)* mutant allele is selected in stochastic environments when thermal stress is frequently encountered

**Figure 3:**
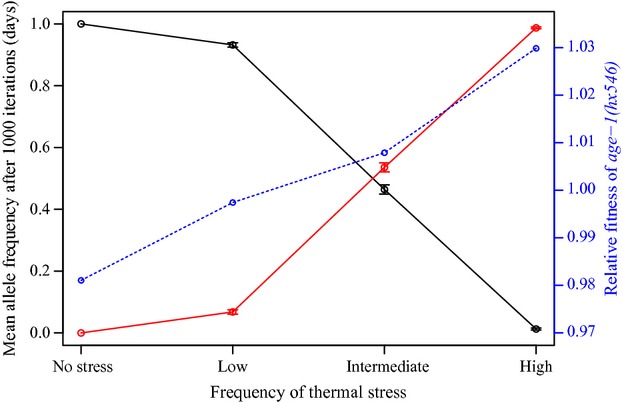
The *age-1(hx546)* mutant allele confers a selective advantage in stochastic environments when thermal stress is frequently encountered. Mean allele frequencies (±SE of the means) of each genotype after 1000 iterations (days), and relative fitness of the *age-1(hx546)* allele (blue line), when food availability varied over time and no thermal stress was imposed and when thermal stress was imposed at low, intermediate and high frequencies. Black lines represent the wild-type *age-1(+)* genotype and red lines represent *age-1(hx546)* mutants. Data correspond to 1000 Monte Carlo projections for each set of environmental conditions. For each set of simulations, the mean number of iterations (±SE) that populations were projected in each environmental state is presented in Table S3).

Having demonstrated that exposure to intense thermal stress can indirectly select for increased longevity when excess food is available, we used stochastic population projection models to examine how selection acts upon the wild-type and *age-1(hx546)* mutant alleles when both food availability and temperature vary over time. Whilst frequencies at which periods of thermal stress (27°C or 30°C) were encountered varied among simulations, the number of iterations with or without food remained approximately equal (Table S3). When only food availability varied over time, *age-1(hx546)* mutants had substantially lower fitness than the wild-type genotype (Fig. 3). A marginal fitness deficit was also observed for *age-1(hx546)* mutants when thermal stress was encountered at low frequencies (Fig. 3). However, *age-1(hx546)* mutants displayed a slight advantage over the wild-type genotype when thermal stress was imposed at intermediate frequencies, and had considerably higher fitness when thermal stress was encountered at high frequencies (Fig. 3). Although these results are based upon a simplified model, in which density-dependent processes and effects of the maternal environment are not taken into account, they imply that the fitness costs observed in *age-1(hx546)* mutants under nutrient-limited conditions may not be important in environments in which additional adverse conditions are frequently encountered.

## Discussion

Throughout evolutionary history, mutations which promote longevity are likely to have arisen in natural populations. Such mutations would likely lead to a disadvantage under most environmental conditions and eventually be purged by selection (Van Voorhies et al. 2006). However, we have demonstrated experimentally that a mutation which increases life span and stress resistance in *C. elegans* can confer a selective advantage when fluctuations in the environment include states that are stressful. This context-dependent gain of fitness in a laboratory-derived mutant may reflect situations in which mutations that increase life span can be maintained and selected in natural populations that experience heterogeneous environmental conditions. The results from the stochastic population projection models further corroborate these findings and imply that increased longevity can evolve when genetic variation in the ability to tolerate stress is present in populations which frequently experience harsh environments, even if long-lived, stress-resistant alleles disrupt the optimal responses to certain conditions.

Our assessment of gene-by-environment interactions provides some insight into the fitness differences that arose between *age-1(hx546)* mutants and the wild-type genotype under different environmental conditions. For example, the increased relative fitness of *age-1(hx546)* mutants in populations which were maintained with unlimited food and periodically exposed to 30°C can likely be explained by the greater survival and fecundity that was observed following exposure to similar environmental conditions. Temperature-induced dauer formation at 27°C (Malone et al. 1996) is likely to be the underlying cause of the delayed maturity observed in *age-1(hx546)* mutants which were exposed to this temperature during the L1 stage. However, dauer formation under these conditions may also have protected the larvae against the damaging effects of high temperatures, making *age-1(hx546)* mutants more fecund than wild-type individuals later in life. The effects of these plastic responses to intermediate temperature stress may counteract one another, explaining why no fitness differences were observed between the genotypes when populations with unlimited food were periodically exposed to 27°C.

The delayed maturity observed in *age-1(hx546)* mutants that had arrested in the dauer stage would lead to a reduction in population growth rates relative to the wild-type genotype on each occasion that starved populations encounter a source of food. This at least partially explains why fitness costs arise in *age-1(hx546)* mutants under nutrient-limited conditions, regardless of the temperature treatment applied. *C. elegans* wild isolates have most often been collected from their natural habitats in the dauer stage (Barrière and Félix 2005), suggesting that populations frequently encounter unfavorable conditions. The ability of *C. elegans* to arrest development in the dauer stage is likely to allow populations to persist in heterogeneous environments which include harsh abiotic conditions, and may negate the requirement for additional stress resistance to evolve. Nevertheless, the results from our stochastic population projection models suggest that when temporal fluctuations in food availability are combined with periodic exposure to high temperature stress, opportunities may arise in which mutations that increase stress resistance and longevity can be favored by selection.

Exposure to environmental stress is considered to be an important factor shaping evolutionary trajectories in wild populations (Hoffmann and Hercus 2000). Stressful conditions may facilitate micro-evolutionary transitions by increasing the rate at which new genotypes arise and by imposing selection pressures that accelerate the rate at which alleles become fixed in a population (Nevo 2001; Wright 2004). However, if trade-offs associated with stress resistance reduce fitness relative to other genotypes in some conditions, stress-resistant alleles are only likely to be favored in certain environments (Hoffmann and Parsons 1991; Partridge et al. 1995; Shirley and Sibly 1999). Consistent with this, intra-specific differences in the ability to tolerate stress among populations from distinct environments co-vary with longevity in several invertebrates (Nevo et al. 1998; Grewal et al. 2002; Lazarevic et al. 2007). This is likely to reflect adaptation to local conditions and may be a consequence of selection acting upon genetic variation in stress response pathways and/or downstream effector genes.

It is currently unclear if natural variation in genes encoding components of the IIS pathway exists within and among *C. elegans* populations. However, several genes which influence stress resistance and longevity in model organisms are polymorphic in natural populations of *Drosophila melanogaster*, and some of these show evidence of adaptive selection across latitudinal clines (Schmidt et al. 2000; Geiger-Thornsberry and Mackay 2004; Williams et al. 2006; Paaby et al. 2010). For example, natural variation in the *D. melanogaster age-1* homologue, *Dp110*, is associated with differences among populations from different latitudes in the propensity to arrest in reproductive diapause (Williams et al. 2006), a trait which is linked to longevity and stress resistance (Tatar et al. 2001). Moreover, in laboratory populations, exposure to low temperature stress drives a shift in allele frequencies which favors diapausing genotypes (Schmidt and Conde 2006). These studies exemplify the importance of temperature as a selection pressure underlying life-history evolution in nature.

Wild populations harbor considerable genetic variation for resistance to thermal stress (Sorenson et al. 2001; Fangue et al. 2006). The existence of such standing genetic variation may be vital for populations to persist during extreme climatic events and/or to adapt to rapid changes in the local environment. Environmental change may lead to modified patterns of thermal stress through changes in daily maximum and minimum temperatures, and, even if mean temperatures do not change, selection upon different life-history strategies may be driven by temporal variance. As life-history traits such as fecundity and dispersal may vary with changes in stress resistance and longevity (Partridge et al. 2005; Hanski et al. 2006), this suggests the potential for important indirect effects on population dynamics.

At the proximate level during an individual lifetime, the potential longevity of stress-resistant genotypes may not be apparent in all environmental conditions (Van Voorhies et al. 2005). Whilst exposure to mild stressors can increase life span, extreme forms of stress are likely to inhibit longevity by disrupting mechanisms which maintain cellular homeostasis (Yu 2004). Nevertheless, our results suggest that, over ecological time scales, heterogeneity in environmental stress can alter the life-history strategy which is optimal for a population and indirectly lead to the evolution of different life spans. Given contemporary transitions in global climate, and additional anthropogenic pressures, this may have important implications for the evolution of life-history strategies.

## Supporting Information

**Figure S1.** Schematic illustration of experimental design. **Figure S2.** Stage/age classes included within each population projection matrix. **Figure S3.** Randomization of matrix sequences. **Table S1.** Mean survival to maturity, times to maturity, and fecundity (± standard errors of the means, SE) after the 24-h stress treatments. **Table S2.** Major population projection matrix events. **Table S3.** Mean number of daily iterations (± standard errors of the means, SE) in each environmental state for each set of 1000 randomized population projections.
