# Supplementary material for: Stressful environments can indirectly select for increased longevity
Source: Ecol Evol. 2014 Mar 10;4(7):1176–85. doi: 10.1002/ece3.1013 (PMC3997331; doi:10.1002/ece3.1013)
Supplement: Supplementary file 1 — Figure S1. Schematic illustration of experimental design. Figure S2. Stage/age classes included within each population projection matrix. Figure S3. Randomization of matrix sequences. Table S1. Mean survival to maturity, times to maturity, and fecundity (± standard errors of the means, SE) after the 24-h stress treatments. Table S2. Major population projection matrix events. Table S3. Mean number of daily iterations (± standard errors of the means, SE) in each environmental state for each set of 1000 randomized population projections. [file ece30004-1176-sd1.docx]

**Supplementary information**

**Figure S1: Schematic illustration of experimental design.** Mixed genotype populations were established with equal proportions of age-synchronised wild type and *age-1(hx546)* mutant L4 larvae. Populations were allowed to grow for several days, then were maintained either with excess food or with limited food for the remainder of the experimental period. Whilst control populations were constantly held at 20°C, stressed populations were periodically exposed to 27°C or 30°C for 24 hours on days 6, 12 and 18. Frequencies of the *age-1(+)* and *age-1(hx546)* alleles were determined on days 6, 12, 18, and 24.

**Figure S2: Stage/age classes included within each population projection matrix.** Solid arrows represent changes which occur during favourable conditions. Dashed arrows represent transitions which occur primarily in starvation conditions (*age-1(hx546)* mutants can also arrest in the dauer stage if exposed to 27°C during early development). Dotted arrows represent transitions which occur during or after exposure to thermal stress. In parentheses, ‘2’ corresponds to individuals which were stressed during stage 2 (L1/L2) and ‘6’ corresponds to individuals which were stressed during stage 6 (L3/L4). Adults which arrested in the reproductive diapause state during exposure to starvation conditions did not progress to subsequent stages because populations were reduced to contain only dauer larvae (the principal dispersal stage) after each starvation event.

**19. Adult (6)**

**16. Adult (6)**

**5. Dauer**

**8. Adult**

**10. Reproductive diapause**

**7. Adult (2)**

**1. Eggs**

**2. L1/L2**

**12. Adult**

**4. L3/L4 (2)**

**9. Adult (6)**

**13. Adult (6)**

**11. Adult (2)**

**3. L1 diapause**

**6. L3/L4**

**15. Adult**

**14. Adult (2)**

**18. Adult**

**17. Adult (2)**

**21. Adult**

**20. Adult (2)**

**Day 1 reproduction**

**Day 2 reproduction**

**Day 3 reproduction**

**Day 4 reproduction**

**Day 5 reproduction**

**22. Adult (6)**

**
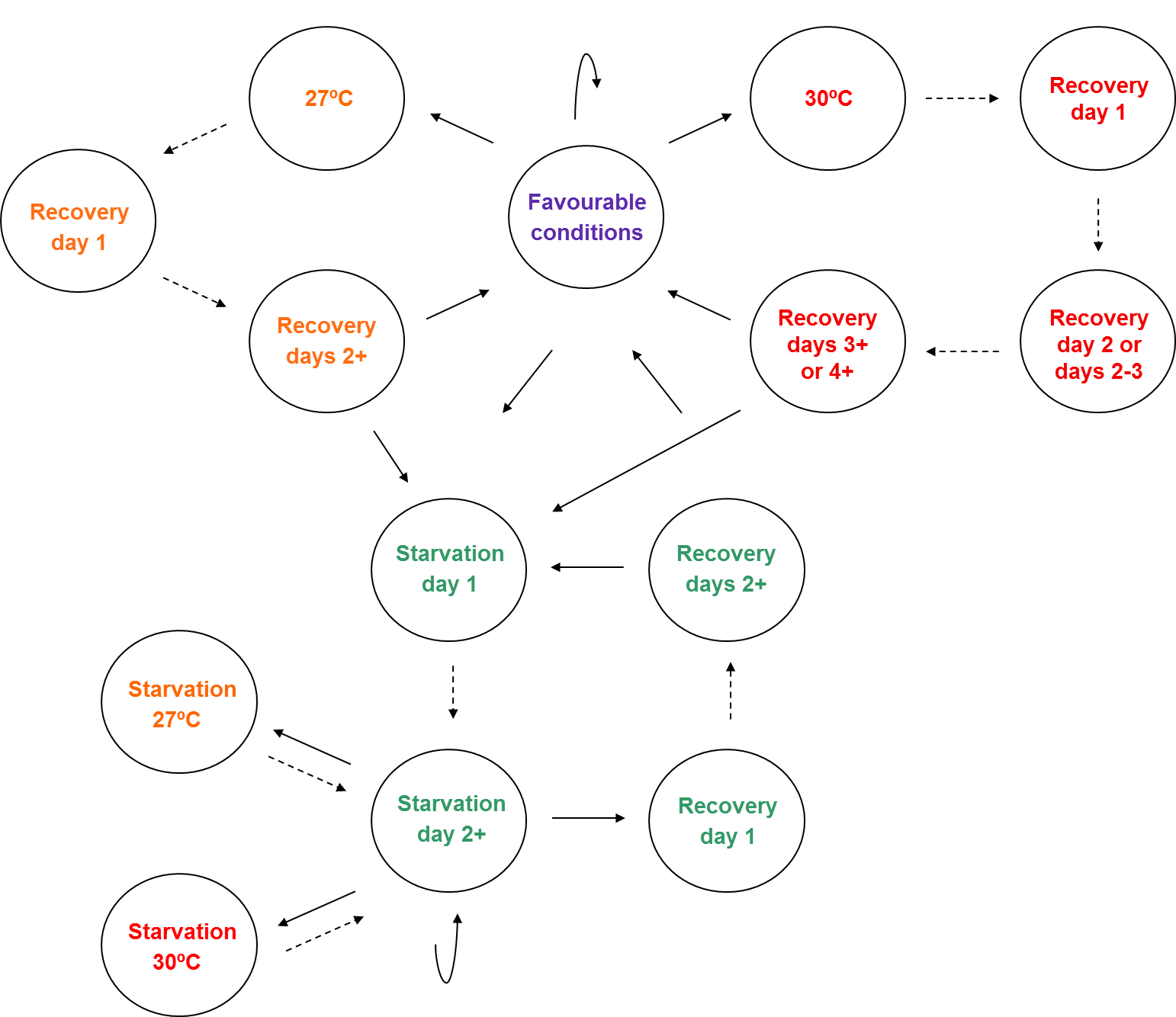
**

**Figure S3: Randomisation of matrix sequences.** Responses to different environmental conditions were represented by 14 projection matrices per genotype (each circle represents a single matrix). New environmental states were selected at random according to a predefined probability (solid arrows). When necessary, populations were projected using a specific sequence of matrices (broken arrows). Matrix sequences were identical for the two genotypes except during recovery from 30°C when food was available (wild type populations were projected with the 2^nd^ recovery matrix for two iterations to account for delayed maturity). During recovery from starvation, populations were projected with the final recovery matrix for at least 2 iterations. This was to ensure that L1/L2s were present in populations to form dauers and prevent extinction if starvation conditions were immediately encountered again. During recovery from thermal stress when food was available, populations were projected with the final recovery matrix for at least 3 iterations to account for the reduced fecundity of individuals which had been stressed during development.

**Table S1: Mean survival to maturity, times to maturity and fecundity (± standard errors of the means, SE) after the 24 hour stress treatments.**

| **Environmental conditions** | | **Stage exposed to stress** | **Mean proportion survival to adulthood ± SE (no. replicates)** | | **Mean time to maturity* (hours) ± SE (no. replicates)** | | **Mean lifetime fecundity ± SE**  **(no. replicates)** | |
| --- | --- | --- | --- | --- | --- | --- | --- | --- |
| **Nutritionstatus** | **Temp.** |  | **N2** | ***age-1 (hx546)*** | **N2** | ***age-1 (hx546)*** | **N2** | ***age-1 (hx546)*** |
| Fed | 20°C | L1 | 1.0 ± 0.0  (100) | 1.0 ± 0.0  (100) | 25.1 ± 0.92  (96) | 25.2 ± 0.85  (97) | 242.28 ± 22.85  (47) | 246.37 ± 24.41  (46) |
| Fed | 20°C | L3 | 1.0 ± 0.0  (100) | 1.0 ± 0.0  (100) | 0.89 ± 0.88  (98) | 1.32 ± 0.86  (100) | 233.85 ± 24.80  (47) | 243.16 ± 30.10  (45) |
| Fed | 27°C | L1 | 1.0 ± 0.0  (99) | 1.0 ± 0.0  (100) | 26.70 ± 1.13  (92) | 41.03 ± 1.16  (59) | 82.87 ± 26.95  (46) | 238.91 ± 25.27  (46) |
| Fed | 27°C | L3 | 1.0 ± 0.0  (100) | 1.0 ± 0.0  (100) | 1.86 ± 0.88  (100) | 2.90 ± 1.12  (100) | 11.16 ± 6.58  (56) | 17.69 ± 7.05  (54) |
| Fed | 30°C | L1 | 0.39 ± 0.04  (100) | 0.89 ± 0.02  (100) | 91.73 ± 3.68  (33) | 67.58 ± 4.30  (73) | 11.20 ± 4.73  (25) | 61.9 ± 16.11  (50) |
| Fed | 30°C | L3 | 0.47 ± 0.02  (100) | 0.92 ± 0.02  (100) | 47.49 ± 6.27  (41) | 34.29 ± 3.69  (69) | 6.43 ± 4.38  (28) | 12.16 ± 7.34  (51) |
| Starved | 20°C | L1 diapause | 1. ± 0.0   (100) | 1. ± 0.0   (100) | 49.48 ± 1.14  (98) | 50.29 ± 1.37  (99) | 234.46 ± 25.48  (50) | 242.20 ± 26.47  (51) |
| Starved | 20°C | Dauer | 1. ± 0.0   (100) | 1. ± 0.0   (100) | 31.17 ± 1.11  (92) | 40.67 ± 0.95  (58) | 242.24 ± 27.76  (50) | 245.58 ± 23.16  (48) |
| Starved | 27°C | L1 diapause | 1. ± 0.0   (100) | 1. ± 0.0   (100) | 50.87 ± 1.08  (100) | 52.27 ± 1.01  (98) | 228.50 ± 21.73  (50) | 229.24 ± 24.06  (51) |
| Starved | 27°C | Dauer | 1. ± 0.0   (100) | 1. ± 0.0   (100) | 31.57 ± 1.39  (81) | 43.07 ± 1.28  (58) | 239.04 ± 23.48  (52) | 241.00 ± 20.29  (49) |
| Starved | 30°C | L1 diapause | 1. ± 0.0   (100) | 1. ± 0.0   (100) | 52.52 ± 1.38  (86) | 53.14 ± 1.49  (72) | 210.60 ± 19.62  (50) | 217.16 ± 19.16  (51) |
| Starved | 30°C | Dauer | 1. ± 0.0   (100) | 1. ± 0.0   (100) | 32.26 ± 1.59  (74) | 43.86 ± 2.13  (76) | 229.63 ± 22.58  (51) | 231.98 ± 20.29  (50) |

*Times for different larval stages to attain maturity after removal from thermal stress do not correspond to the age at maturity because development had started before the larvae were monitored.

**Table S2: Major population projection matrix events**

| **Matrix** | **Environment** | **Wild type (N2)** | ***age-1(hx546)* mutants** |
| --- | --- | --- | --- |
| 1 | Favourable conditions | Optimal growth and reproduction. | As wild type. |
| 2 | Starvation: Day 1 | Eggs (1) hatch and larvae arrest in L1 diapause (3). L1/L2s (2) arrest as dauers (5). L3/L4s arrest in adult reproductive diapause^†^ (10). Adults continue to produce eggs but have reduced fecundity (estimated as 10% of fecundity in favourable conditions). | As wild type. |
| 3 | Starvation: Day 2+ | 25% of eggs (1) hatch externally and arrest in L1 diapause (3). 75% of eggs (1) hatch internally as L1/L2s (2) then arrest in the dauer stage (5) on the 2^nd^ iteration. Reproductive adults die due to internal hatching. | As wild type. |
| 4 | Recovery from starvation: Day 1 | Population growth is initiated only by a proportion (randomised from 0.1-1%, but same for both genotypes at a given time) of dauer larvae. 70% of surviving dauers (5) mature as adults (8), 30% remain arrested. | Population growth is initiated only by a proportion (randomised from 0.1-1%, but same for both genotypes at a given time) of dauer larvae. 30% of surviving dauers (5) mature as adults (8), 70% remain arrested. |
| 5 | Recovery from starvation: Day 2+ | Remainder of surviving dauers (5) mature as adults (8). Populations were projected for at least 2 iterations with this matrix to ensure that L1/L2s (2) were present to reach the dauer stage if starvation conditions were immediately encountered again (otherwise populations would go extinct). | As wild type. |
| 6 | 27°C with excess food | 50% of eggs (1) are non-viable (estimated), L1/L2s (2) progress to post-stress L3/L4s (4). L3/L4s (6) progress to post-stress adults (9). Adults have reduced fecundity (estimated as 5% of fecundity in favourable conditions). Reproductive adults progress to post-stress adult stages (13, 16, 29 or 22 depending on current stage). | As wild type, except L1/L2s (2) progress to the dauer stage (5). |
| 7 | Recovery from 27°C with excess food: Day 1 | Post-stress L3/L4s (4) progress to post-stress adults (7). Post-stress fecundity was reduced to 4.4%* for individuals stressed during the L3/L4 stage (6). | 70% of dauers (5) remain arrested and 30% mature as adults (8). Post-stress fecundity was reduced to 6.9%* for individuals stressed during the L3/L4 stage (6). |
| 8 | Recovery from 27°C, with excess food: Day 2+ | Post-stress fecundity was reduced to 36%* for individuals stressed during the L1/L2 stage (2). Populations were projected for at least 3 iterations with this matrix to account for reduced fecundity in post-stress adults. | Remaining dauers (5) mature as adults (8). Populations were projected for at least 3 iterations with this matrix to account for reduced fecundity in post-stress adults. |
| 9 | 30°C with excess food | All eggs (1) are non-viable (estimated). 60%* of L1/L2s (2) die and 50%* of L3/L4s (6) die. Surviving larvae arrest development and all reproductive adults die (as these are unlikely to contribute further to population growth after the stress period). | All eggs (1) are non-viable (estimated). 10%* of L1/L2s (2) die and 10%* of L3/L4s (6) die. Surviving larvae arrest development and all reproductive adults die (as these are unlikely to contribute further to population growth after the stress period). |
| 10 | Recovery from 30°C with excess food: Day 1 | To account for the post-stress delay in maturity, all surviving larvae remain arrested. | Surviving L1/L2s (2) remain arrested. 20% of L3/L4s (6) mature as post-stress adults (9). |
| 11 | Recovery from 30°C with excess food: Days 2 & 3 (wild type) or Day 2 only (*age-1(hx546)* mutants) | L1/L2s (2) progress to post-stress L3/L4s (4). L3/L4s (6) mature as post-stress adults (9). Fecundity was reduced to 2.5%* in individuals stressed as L3/L4s (6). Populations were projected for 2 iterations with this matrix to account for the delayed maturity in individuals stressed as L1/L2s (2). | L1/L2s (2) progress to post-stress L3/L4s (4). Remaining L3/L4s (6) mature as post-stress adults (9). Fecundity was reduced to 4.8%* in individuals stressed as L3/L4s (6). |
| 12 | Recovery from 30°C with excess food: Days 4+ (wild type) or Days 3+ (*age-1(hx546)* mutants) | Post-stress L3/L4s (4) mature as post-stress adults (7). Fecundity was reduced to 4.8%* for individuals stressed as L1/L2s (2). Populations were projected for at least 3 iterations with this matrix to account for reduced fecundity in post-stress adults. | Post-stress L3/L4s (4) mature as post-stress adults (7). Fecundity was reduced to 26.5%* for individuals stressed as L1/L2s (2). Populations were projected for at least 4 iterations with this matrix to account for reduced fecundity in post-stress adults. |
| 13 | 27°C, starvation^‡^ | Populations contain only the L1 diapause stage (3), dauer larvae (5) and adults in reproductive diapause^†^ (10). All individuals survive and remain arrested. | As wild type. |
| 14 | 30°C, starvation^‡^ | Populations contain only the L1 diapause stage (3), dauer larvae (5) and adults in reproductive diapause^†^ (10). All individuals survive and remain arrested. | As wild type. |

*Genotype differences in survival and fecundity were extrapolated from the values presented in Supplementary Table 1.

^†^Reproductive diapause is a state of arrest formed after larvae experience starvation conditions during the L4 stage (Angelo & Van Gilst. 2009. *Science* 326(5955): 954-958). We assumed that all individuals in reproductive diapause survive exposure to thermal stress because wild type adults have increased resistance to thermal stress when starved (Kaeberlein *et al*. 2006. [*Aging*](javascript:AL_get(this,%20'jour',%20'J%20Gerontol%20A%20Biol%20Sci%20Med%20Sci.');) *Cell* 5: 487-494).

^‡^Recovery matrices were not required following exposure to thermal stress in starvation conditions because populations were subsequently projected using the matrix for starvation day 2+ for at least 1 iteration.

Numbers in parenthesis correspond to stages represented in Supplementary Figure 2. When it was necessary to estimate matrix parameter values, estimates were identical for the two genotypes.

**Table S3: Mean number of daily iterations (± standard errors of the means, SE) in each environmental state for each set of 1000 randomised population projections.**

| **Stochastic environment** | **Mean number of iterations (± SE) per state** | | | | | | **Mean number of dispersal events***  **(± SE)** |
| --- | --- | --- | --- | --- | --- | --- | --- |
|  | **Fed 20°C** | **Fed 27°C** | **Fed 30°C** | **Starved 20°C** | **Starved 27°C** | **Starved 30°C** |  |
| **Resource fluctuation only** | 497.3 ± 0.98 | 0 | 0 | 502.7 ± 0.98 | 0 | 0 | 57.8 ± 0.11 |
| **Low frequency stress** | 488.9 ± 1.09 | 5.4 ± 0.07 | 5.3 ± 0.06 | 489.3 ± 1.11 | 5.6 ± 0.08 | 5.6 ± 0.06 | 56.6 ± 0.13 |
| **Intermediate frequency stress** | 474.1 ± 0.87 | 12.5 ± 0.10 | 12.5 ± 0.09 | 476.9 ± 0.88 | 12.2 ± 0.11 | 12.2 ± 0.09 | 58.3 ± 0.11 |
| **High frequency stress** | 458.4 ± 0.87 | 19.4 ± 0.13 | 19.3 ± 0.11 | 461.8 ± 0.84 | 20.7 ± 0.15 | 20.7 ± 0.11 | 57.0 ± 0.11 |

* Dispersal events correspond to the number of iterations in which population growth was initiated only by dauer larvae following periods of starvation.
